# Characterization of Cellulose-Degrading Bacteria Isolated from Silkworm Excrement and Optimization of Its Cellulase Production

**DOI:** 10.3390/polym15204142

**Published:** 2023-10-19

**Authors:** Hao Li, Minqi Zhang, Yuanhao Zhang, Xueming Xu, Ying Zhao, Xueping Jiang, Ran Zhang, Zhongzheng Gui

**Affiliations:** 1 College of Biotechnology, Jiangsu University of Science and Technology, Zhenjiang 212100, China; lhoscarlh@just.edu.cn (H.L.);; 2 Sericulture Resources Intensive Processing Laboratory, Sericulture Research Institute, Chinese Academy of Agricultural Sciences, Zhenjiang 212100, China

**Keywords:** silkworm excrement, cellulose-degrading bacteria, *Bacillus subtilis*, cellulase, bioaugmentation

## Abstract

An abundance of refractory cellulose is the key limiting factor restricting the resource utilization efficiency of silkworm (*Bombyx mori*) excrement via composting. Screening for cellulose-degrading bacteria is likely to provide high-quality strains for the safe and rapid decomposition of silkworm excrement. In this study, bacteria capable of degrading cellulose with a high efficiency were isolated from silkworm excrement and the conditions for cellulase production were optimized. The strains were preliminarily screened via sodium carboxymethyl cellulose culture and staining with Congo red, rescreened via a filter paper enzyme activity test, and identified via morphological observation, physiological and biochemical tests, and phylogenetic analysis of the 16S rDNA sequence. Enzyme activity assay was performed using the 3,5-dinitrosalicylic acid method. DC-11, a highly cellulolytic strain, was identified as *Bacillus subtilis*. The optimum temperature and pH of this strain were 55 °C and 6, respectively, and the filter paper enzyme activity (FPase), endoglucanase activity (CMCase), and exoglucanase activity (CXase) reached 15.40 U/mL, 11.91 U/mL, and 20.61 U/mL. In addition, the cellulose degradation rate of the treatment group treated with DC-11 was 39.57% in the bioaugmentation test, which was significantly higher than that of the control group without DC-11 (10.01%). Strain DC-11 was shown to be an acid-resistant and heat-resistant cellulose-degrading strain, with high cellulase activity. This strain can exert a bioaugmentation effect on cellulose degradation and has the potential for use in preparing microbial inocula that can be applied for the safe and rapid composting of silkworm excrement.

## 1. Introduction

A large amount of agricultural waste is produced in China each year, with plant organic waste (mainly comprising cellulose) accounting for 80% of the waste. Cellulose is a polymer with several glucose units linked via β-1,4-glycosidic bonds that give plant cell walls their rigid structure. Cellulose is the most common element in lignocellulosic biomass bioresources [1]. The inappropriate use of cellulose-rich waste leads to resource loss and is not environmentally friendly. Therefore, the need to achieve its effective use has become a longstanding issue. Currently, composting is considered to be the main method for the recycling of such cellulose waste [2]. However, the abundance of refractory cellulose is a bottleneck restricting the efficient recycling of agricultural waste, which seriously limits the composting efficiency of such waste.

Cellulases produced by microorganisms such as bacteria and fungi play the most important role in cellulose biodegradation. The process of cellulose biodegradation is a complex enzymatic reaction in which cellulose is degraded into glucose units via the action of various cellulases. Cellulases can be classified into endo-β-1,4-glucanase, exo-β-1,4-glucanase, and β-1,4-glucosidase [3,4]. High-yield cellulase-degrading bacteria are often used as exogenous microbial agents and are widely employed for treating cellulose-rich agricultural waste. These bacteria exhibit the advantages of being environmentally friendly, cheap, convenient, and not causing secondary pollution. Therefore, the screening of functional strains with high cellulase production has attracted immense attention. Egwuatu et al. reported the isolation of 20 cellulose-degrading bacteria from sheep rumen, the enzyme activity of which ranged from 0.225 U/mL to 1.652 U/mL [5]. Harnvoravongchai et al. isolated and characterized thermophilic anaerobic bacteria with cellulose- and hemicellulose-degrading activities from a tropical dry deciduous forest in northern Thailand [6]. Kim et al. isolated a cellulose-degrading bacterium from the sediment in a branch of the Nackdong River in Sangju, South Korea [7]. However, the cellulase activity of the isolated strains has generally been low, and even those with a high enzyme activity have been unstable in subsequent cultures. Therefore, the screening of strains with high cellulase activity from different environments and optimizing their enzyme-producing properties continues [8].

The richness in refractory cellulose is the bottleneck restricting the efficient recycling of agricultural wastes such as silkworm excrement. Silkworm excrement is the collective name for the excrement and leftover mulberry pulp excreted by silkworms, which is a type of multi-component material rich in crude protein and cellulose. The cellulose content of silkworm excrement has been reported to be as high as 20%. Several studies have shown that cellulose-degrading functional bacteria isolated from cellulose-rich waste environments can considerably accelerate the degradation of cellulose, improve the conversion efficiency of agricultural waste composting, and shorten the process of composting [3]. Therefore, the isolation of new high-yielding cellulase-producing functional bacteria from cellulose-rich waste environments and the study of their ability to produce enzymes and degrade cellulose will help to obtain excellent strains that can degrade cellulose efficiently. This is extremely important for the research and development of microbial inoculants for the safe treatment of cellulose-rich agricultural waste such as silkworm excrement [9,10]. 

This study aims to screen novel high-efficiency cellulose-degrading bacteria from silkworm excrement for treatment of cellulose-rich agricultural waste. Firstly, the isolates are preliminarily screened using the sodium carboxymethyl cellulose (CMC-Na) culture method and staining with Congo red, followed by rescreening with a filter paper enzyme activity test. Then, the novel high-efficiency cellulose-degrading bacteria are identified via morphological observation, physiological and biochemical tests, and phylogenetic analysis of the 16S rDNA sequence. The culture conditions for the novel high-efficiency cellulose-degrading bacteria will be optimized to achieve maximum cellulase production. Additionally, bioaugmentation of silkworm excrement with the novel high-efficiency cellulose-degrading bacteria will be also performed. The findings are likely to be helpful for the commercial application of the strain for the bioconversion of cellulose and the safe treatment of cellulose-rich agricultural waste.

## 2. Materials and Methods

### 2.1. Culture Media and Sample Collection

Luria–Bertani (LB) medium was used for bacterial growth (10 g·L^−1^ peptone, 5 g·L^−1^ yeast extract, 10 g·L^−1^ NaCl). Carboxymethyl cellulose (CMC) medium (10 g·L^−1^ CMC-Na, 1 g·L^−1^ K_2_HPO_4_, 1.0 g·L^−1^ NH_4_NO_3_, 0.02 g·L^−1^ CaCl_2_, 0.2 g·L^−1^ 7H_2_O·MgSO_4_, 0.05 g·L^−1^ 6H_2_O·FeCl_3_) was used for the screening and isolation of cellulolytic bacteria. The cellulose-degrading ability of bacterial isolates was confirmed with the Congo red agar medium (2 g·L^−1^ cellulose, 2 g·L^−1^ gelatin, 0.5 g·L^−1^ KH_2_PO_4_, 0.25 g·L^−1^ MgSO_4_, 15 g·L^−1^ agar, and 0.2 g·L^−1^ Congo red). LB-CMC liquid medium (10.0 g·L^−1^ tryptone; 5.0 g·L^−1^ yeast extract; 10.0 g·L^−1^ NaCl and 5 g·L^−1^ CMC-Na, 50 mL, pH 7.0) was used for the optimization of cellulase production. All chemicals with high purity grade (>90%), including CMC-Na, K_2_HPO_4_, peptone, and other compounds, were obtained from Sinopharm Co., Ltd. (Beijing, China) and Sangon Co., Ltd. (Shanghai, China). All media were sterilized at 121 °C for 30 min before use. The silkworm excrement was taken from a silkworm excrement septic tank of the sericulture base at the Sericulture Research Institute, Chinese Academy of Agricultural Sciences, Zhenjiang City, Jiangsu Province, China. The freshly collected silkworm excrement samples were stored at 4 °C for later use [11].

### 2.2. Primary Screening of Cellulolytic Bacteria

Silkworm excrement samples (5 g) were weighed and added to 100 mL of CMC liquid medium with CMC-Na as the only carbon source [12]. The enrichment culture was placed in a shaker at 30 °C and 180 rpm for 30 min, after which approximately 4 mL of the mixture was transferred to a flask containing fresh medium and shaken for a further 72 h. After performing enrichment culture three times, the bacterial solution was diluted to 10^−3^, 10^−4^, and 10^−5^. The samples were plated on LB agar plates at 30 °C for 72 h, and colonies with different phenotypes (size, color, and shape) were isolated [11]. Ten-fold dilutions of the suspension of well-isolated colonies were spread on CMC-Congo red plates and incubated at 30 °C for 48 h. The hydrolyzing capacity (*HC*) of the cellulose Congo red agar was calculated from the ratio of the halo band to bacterial colony diameter (D/d). The hydrolytic activity of each isolate was determined based on the *HC* value [13]. 

The cellulolytic index (*HC*) was calculated as follows:

(1) $$HC=\;\frac{D}{d}$$

*D*—Diameter of hydrolysis circle, cm;

*d*—Diameter of colony, cm.

### 2.3. Rescreening of Cellulolytic Bacteria

The filter paper disintegration test was used as a verification method for the rescreening of cellulolytic bacteria to observe the disintegration ability of the strains on filter paper [14]. The spore suspension of the primary screening strain was centrifuged and washed twice with 1× M9 buffer and then inoculated into the medium with filter paper (starch-free filter paper, 2 × 5 cm, three samples per bottle) as the only carbon source. Inactivated bacterial solution that was prepared via high temperature and high pressure was added as the control group (CG), and the culture was incubated in a shaker at 37 °C and 200 rpm for 72 h. The disintegration of the filter paper was observed and recorded every 24 h [14]. The decomposed filter paper of the experimental group and the completely intact filter paper of the CG were removed with tweezers and sampled with the ethanol gradient dehydration method that involved dehydration with ethanol using a gradually increasing concentration gradient (20% volume fraction gradually increased to 100%). The dehydration time decreased with increasing volume, from 30 min to 3–4 min. Finally, after freeze-drying for 24 h, the filter paper was sprayed with gold for sample preparation [15]. The surface morphology of the filter paper was observed with field emission scanning electron microscopy (FE-SEM S-4800, Hitachi, Tokyo, Japan). Finally, strains with high cellulolytic index and those with clear disintegration of the filter paper were selected for purification [5]. 

### 2.4. Quantification of Cellulase Activity

Cellulase activities were determined quantified using the 3,5-dinitrosalicylic acid (DNS) assay method as recommended by the International Union of Pure and Applied Chemistry (IUPAC) Commission on Biotechnology [16]. For quantifying endoglucanase activity (CMCase), 1 mL of the supernatant of the cellulolytic bacteria (crude enzyme solution) was taken and equalized with 2 mL of DNS reagent and 1 mL of 1% CMC-Na (pH 4.5) substrate in a water bath at 50 °C for 60 min. The content was then heated in a boiling water bath for 5 min. The absorbance was read at 540 nm using an ultraviolet UV spectrophotometer (Systronics 2202 PC double beam spectrophotometer). CMCase activity was calculated using the inactivated enzyme as the blank control. The amount of reducing sugars was determined using the standard graph of glucose. One unit of enzyme activity was defined as the amount of enzymes liberating 1 µmol of reducing sugars in the form of glucose per minute under the assay conditions. For quantifying exoglucanase activity (CXase), 1 mL of the supernatant (crude enzyme solution) and 1 mL of 1% avicelase (pH 4.5) substrate were placed in a water bath at 50 °C for 60 min, after which the reaction was terminated via adding 2 mL of DNS reagent. Furthermore, the contents were heated in a boiling water bath for 5 min. The tubes were allowed to cool and the absorbance was read at 540 nm in a UV spectrophotometer. CXase activity was calculated using the inactivated enzyme as the blank control. The amount of enzyme producing 1 μmol of glucose per minute under the assay conditions was defined as 1 U. For quantifying the enzyme activity (FPase) against the filter paper, 1 mL of the supernatant (crude enzyme solution) and 0.1 g of the filter paper were prepared and 1 mL of pH 4.5 buffer solution was added to a 50 °C water bath for 60 min. After cooling for 5 min at 50 °C, the release of reducing sugars was determined using a UV spectrophotometer at 540 nm. The FPase activity was calculated using the inactivated enzyme as the blank control. The amount of enzyme producing 1 μmol of glucose per minute under the assay conditions was defined as 1 U [17]. Regarding the definition of enzyme activity, under certain temperature and pH conditions, the amount of enzyme required to produce 1 μmol of glucose from the substrate in 1 min was expressed as one unit of enzyme activity in the form U/mL. For the cellulase activity assay, the enzyme activity was calculated in accordance with the IUPAC method using the following equation:

(2) $$\mathrm{Enzyme}\;\mathrm{activity}\;(U/\mathrm{mL})=\;\frac{C\times N\times 1000}{Vs\times t}$$

*C*—glucose production, mg;

*N*—diluted multiples;

*Vs*—enzyme dosage, mL;

*t*—reaction time, h.

### 2.5. Identification of Cellulolytic Bacteria and Growth Curve

The isolate with the best cellulose-degrading ability was identified via morphological, biochemical, and 16S rDNA sequence analyses. Gram staining was used to characterize the shape of the isolate and determine whether it was Gram-positive or -negative. Basic biochemical tests, such as the indole test, hydrogen sulfide production test, gelatin liquefaction test, starch hydrolysis test, contact enzyme assay, methyl-red test, Voges Proskauer (VP) test, and salt tolerance test were also performed to identify the bacteria at the genus level [18]. The total DNA of the isolate was extracted using the Bacterial Genomic DNA Mini Kit (Sangon, Shanghai, China) after culture enrichment. Universal primers 27F (5′-AGAGTTTGATCCTGGCTCAG-3′) and 1492R (5′-TACGGTTACCTTGTTACGACTT-3′) were used to amplify a part of the 16S rRNA gene via the polymerase chain reaction (95 °C for 3 min, followed by 30 cycles at 95 °C for 30 s, 55 °C for 30 s, 72 °C for 45 s and a final extension at 72 °C for 10 min). After DNA sequencing of PCR products, the obtained sequences were deposited in the NCBI Sequence Read Archive under accession number ON724007. The nucleotide sequences were used as queries for searches in NCBI using the BLAST search function. Phylogenetic trees for subsequent analysis were constructed with the neighbor-joining method using MEGA5 [19]. A stock solution of the isolate (200 μL) was inoculated in 100 mL of LB liquid medium, and *OD*_600_ was measured every 2 h for 48 h using a microplate reader (M200pro; Tecan, Shanghai, China) after incubation at 37 °C and 180 rpm. The growth curve of the strain was plotted by determining the average value obtained from triplicate samples [20].

### 2.6. Optimization of Cellulase Production

Cellulase production was optimized via varying the parameters such as incubation time, inoculum concentration, temperature, and pH. An Erlenmeyer flask with a volume of 250 mL was used for all microbial cultures. To determine the optimal inoculum time, the LB-CMC liquid medium containing a bacterial strain cultured overnight (500 µL, OD approximately1.5) was incubated at 220 rpm. The effect of incubation time on enzyme production was quantified through collecting culture samples at different time points (4, 12, 20, 24, 36, and 48 h) [21]. The effect of inoculum concentration on enzyme production following scheme was used: Six different inocula (1%, 2%, 3%, 4%, 5% and 6%, *v*/*v*) were inoculated in 50 mL of LB-CMC liquid medium on a rotary shaker at 220 rpm for 24 h. Under the optimum inoculum concentration, seven different initial pH values (3, 4, 5, 6, 7, 8, 9, and 10) of the LB-CMC liquid medium were selected to optimize the cellulase production of the isolate. The inoculum was inoculated in 50 mL of LB-CMC liquid medium and cultured in a rotary shaker at 220 rpm and 37 °C for 24 h. The LB-CMC liquid medium (50 mL) containing the bacterial strain cultured overnight (500 µL) was placed in a shaking incubator (220 rpm) at 30, 40, 50, 55, 60, 65, and 70 °C for 24 h. The effect of temperature on enzyme production was investigated at the optimum inoculum concentration and pH [22,23]. The activities of the three cellulases (CMCase, CXase, and FPase) in all samples were measured to identify the optimal conditions.

### 2.7. Bioaugmentation of Silkworm Excrement with DC-11

The screened strain with the best cellulolytic ability (DC-11) was inoculated into a 500 mL Erlenmeyer flask containing 300 mL of LB medium, and cultured at 37 °C for 12 h. The cultured LB medium was centrifuged at 10,000× *g* and 4 °C to separate the bacteria. Cells collected after centrifugation were suspended in phosphate buffered saline and diluted to 10^8^ CFU/mL in distilled water for further use [24]. A total of 800 g of fresh silkworm excrement was added to 2 L polypropylene containers. The treatment groups (TGs, those treated with the bacterial strain) were inoculated with 20 mL of distilled water containing 10^8^ CFU/mL of bacterial cells, whereas the control groups (CGs) were inoculated with 20 mL of distilled water without bacterial cells [25]. The excrement and bacteria were homogenized in each container. Each treatment included three replicates, which were maintained in a greenhouse at 27 °C with 60–70% relative humidity. Samples were taken on days 0 and 6, and the cellulose content in the silkworm excrement was determined using a previously reported method [16,26]. The structural changes in silkworm excrement were studied with a scanning electron microscope (HitachiS-4800, Tokyo, Japan) operated at an acceleration voltage of 3.0 kV. 

### 2.8. Statistical Analysis

One-way analysis of variance was performed using the Statistical Package for the Social Sciences (SPSS 22.0) to determine the significance of differences within different indexes. Tukey’s test was applied to the data. All experiments were performed in triplicate. Differences were considered significant at *p* < 0.05.

## 3. Results

### 3.1. Isolation and Screening of Cellulolytic Bacteria

**Figure 1:**
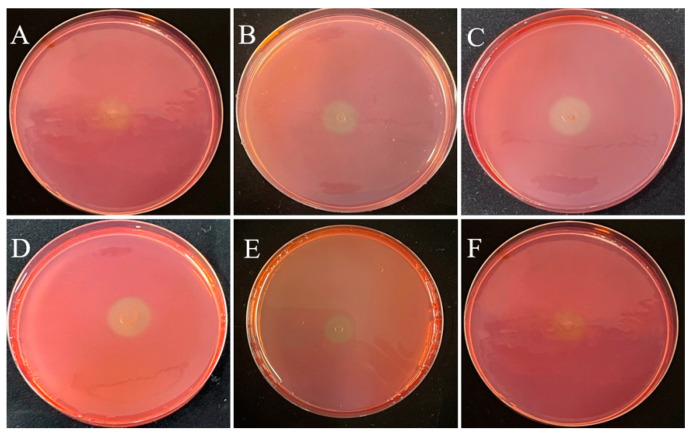
Zone of clearance on cellulose Congo red agar plates for isolates DC-5 (**A**), DC-10 (**B**), DC-11 (**C**), DC-20 (**D**), DC-25 (**E**), and DC-28 (**F**) after 48 h of incubation.

**Figure 2:**
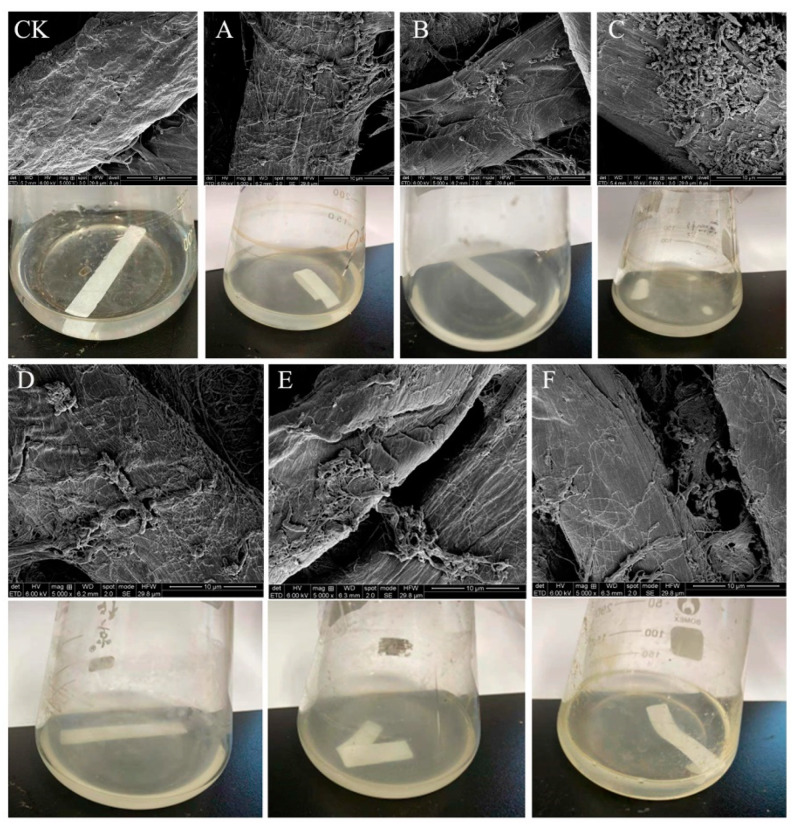
FE-SEM and morphology of filter paper treated with the isolates. (**CK**, Control Check)…. The filter paper degraded by strains DC-5 (**A**), DC-10 (**B**), DC-11 (**C**); DC-20 (**D**); DC-25 (**E**); and DC-28 (**F**) for 48 h. Filter paper with no isolate added was used as the control (**CK**).

This study was performed to isolate potential cellulolytic bacteria from silkworm excrement for the composting of agricultural waste. The Congo red plate method, which is cheap and convenient to perform, is an important tool for the screening of cellulolytic strains [27]. In this study, 35 bacterial isolates were cultured on LB medium, of which 6 isolates (DC-5, DC-10, DC-11, DC-15, DC-25, and DC-28) exhibited cellulose-degrading activities and showed positive results on the Congo red plate (Figure 1). The zone of clearance produced by the individual microbes was measured, the results of which are presented in Table 1. The cellulolytic index ranged from 2.30 to 3.50, which indicated robust CMCase production. Of the six different strains, DC-11 had the highest cellulolytic index (3.50), whereas DC-25 had the lowest (2.30). These findings signify that the bacterial strains obtained from silkworm excrement could be employed for cellulose degradation. In addition, as shown in Figure 2, all six isolates were able to degrade the filter paper. Turbidity formed in the test tube post filter paper decomposition. Cellulose degradation was particularly pronounced in the filter paper treated with DC-11 (Figure 2C), which indicated that DC-11 had the strongest degradation ability. These findings were confirmed using the results of FE-SEM analysis of the treated filter paper [28]. 

### 3.2. Quantification of Cellulase Activity for the Rescreening of Cellulolytic Bacteria

**Figure 3:**
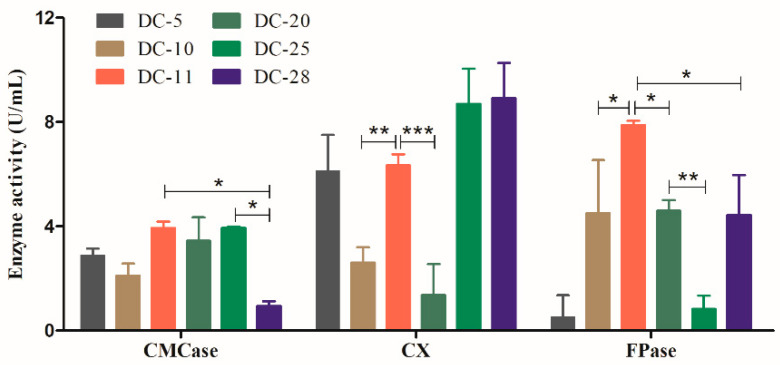
Extracellular cellulase (CMCase, CXase, and FPase) activity of all isolates. Data were calculated as mean ± SD from triplicate measurements. Statistical analysis was performed using one-way analysis of variance (* *p* ≤ 0.05, ** *p* ≤ 0.01, *** *p* ≤ 0.001).

To further identify the bacterial isolates with the strongest cellulolytic ability, the cellulase activities of the six strains were measured. Cellulase assays were performed based on the ability of the individual isolates to hydrolyze cellulose to reducing sugars. In this study, the activities of the three cellulases were determined using the DNS assay method. As shown in Figure 3, CMCase and FPase activities of DC-11 were significantly higher than those of other strains (*p* < 0.05). The CXase activity of DC-11 was significantly higher than those of DC-10 and DC-20 (*p* < 0.01), but did not differ significantly from those of DC-5, DC-25, and DC-28. The CMCase and CXase activities of DC-11 reached 3.26 ± 0.59 and 6.33 ± 0.61 U/mL, respectively, and its FPase activity was as high as 7.87 ± 0.25 U/mL. The above results show that DC-11 has the strongest cellulase-producing ability among the newly isolated strains and can efficiently degrade cellulose.

### 3.3. Identification of the Isolated Cellulolytic Bacteria

**Figure 4:**
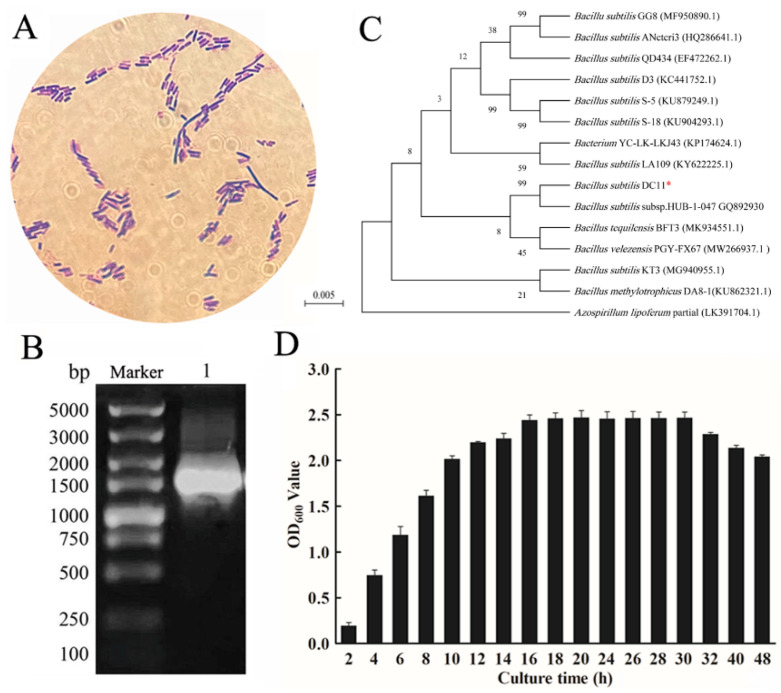
Identification of strain DC-11. Gram staining (**A**), phylogenetic tree (**B**), agarose gel electrophoresis of 16S rDNA (**C**), and growth curve (**D**) of strain DC-11. *: Isolated DC-11 in this study.

The bacterial isolate DC-11 with a high cellulase yield was identified based on its morphological, biochemical, and physiological characteristics, and 16S rRNA gene sequence. Morphological analysis was performed based on the Gram staining method. Gram staining and microscopic examination showed that the isolated strain DC-11 was Gram-negative and rod-shaped (Figure 4A). The results of the biochemical and physiological analyses are presented in Table 2. Strain DC-11 was positive for the following physiological and biochemical tests: hydrogen sulfide production, gelatin liquefaction, contact enzyme assay, indole production, V-P test, salt tolerance test, and starch hydrolysis test. The only test for which a negative result was obtained was the methyl red test. The result of agarose gel electrophoresis of the 16S rDNA sequence of strain DC-11 is depicted in Figure 4B. The DNA fragments amplified via PCR were single bands, and their length was approximately 1500 bp. The PCR products of strain DC-11 were sequenced to obtain the full-length 1492 bp gene sequence, which agreed with the electrophoresis results. The results of the 16S rRNA gene sequencing were used to construct the phylogenetic tree of strain DC-11 (Figure 4C). Phylogenetic analysis revealed that the strain most closely related to DC-11 was *B. subtilis* subsp. HUB-1-047 (99% 16S rDNA sequence similarity). Combined with the physiological and biochemical results, DC-11 was identified as *B. subtilis* and named as *B. subtilis* DC-11.

### 3.4. Optimization of Conditions for Cellulase Production by Strain DC-11

**Figure 5:**
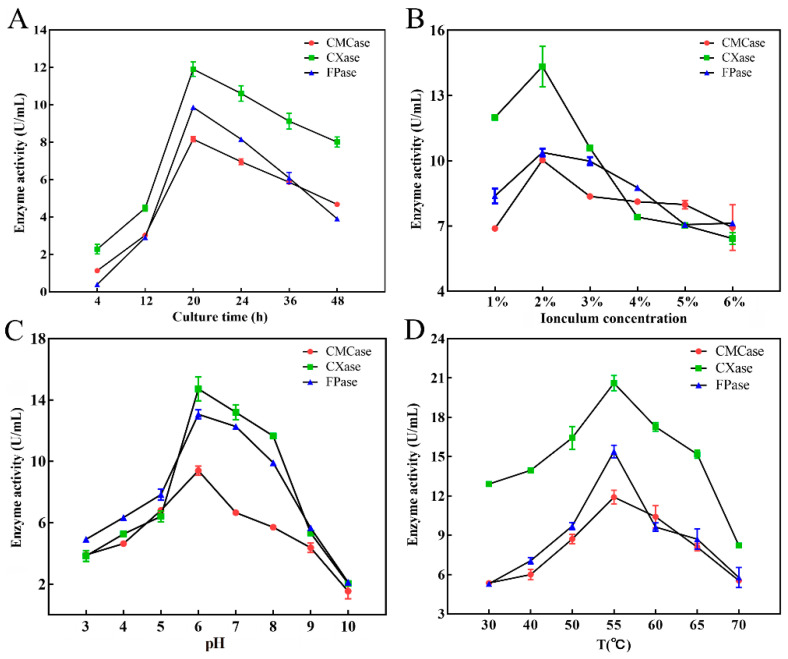
Effects of culture time (**A**), inoculum concentration (**B**), pH (**C**), and temperature (**D**) on cellulase production of strain DC-11.

To optimize conditions for cellulase production using DC-11, its growth curve was first plotted. This strain reached the logarithmic growth phase at 8–16 h after inoculation, and its growth plateaued after 20 h (Figure 4D). The results showed that incubation time, inoculum concentration, temperature, and pH had significant effects (*p* < 0.05) on cellulase production (Figure 5). The effect of incubation time on cellulase activity is illustrated in Figure 5A. As the culture time increased, the cellulase activity first increased and then decreased. When the culture time was 20 h, activities of the CMCase, CXase, and FPase peaked at 8.16, 11.91, and 9.86 U/mL, respectively. The effect of the inoculum concentration of DC-11 on cellulase activity also showed a trend of first increasing and then decreasing. Maximum degradation of cellulose occurred when the inoculum concentration was 2%, at which the CMCase, CXase, and FPase activities reached 10.03, 14.34, and 10.38 U/mL, respectively (Figure 5B). Optimum pH and temperature are key factors for cellulase production. The enzyme production curve of strain DC-11 at various initial pH values is shown in Figure 5C. All three cellulase activities reached their maximum at pH 6; at this pH, the CMCase, CXase, and FPase activities reached 8.95, 14.88, and 12.97 U/mL, respectively. The results indicated that the optimum pH of DC-11 for cellulase production was 6. Furthermore, all three cellulases remained highly active over a broad range of pH values (from 4.0 to 9.0). The effect of different temperatures on the enzymatic activity was evaluated at the optimum pH, incubation time, and inoculum concentration, the results of which are presented in Figure 5D. Strain DC-11 was able to tolerate the temperature range of 30–70 °C. The optimum temperature for the production of cellulase by DC-11 was 55 °C, which indicates that DC-11 is a thermophilic cellulolytic bacterium. At the optimum inoculum concentration, pH, and temperature, the activities of CMCase, CXase, and FPase reached up to 11.91, 20.61, and 15.40 U/mL, respectively. 

### 3.5. Bioaugmentation with DC-11 Enhanced the Degradation of Silkworm Excrement 

**Figure 6:**
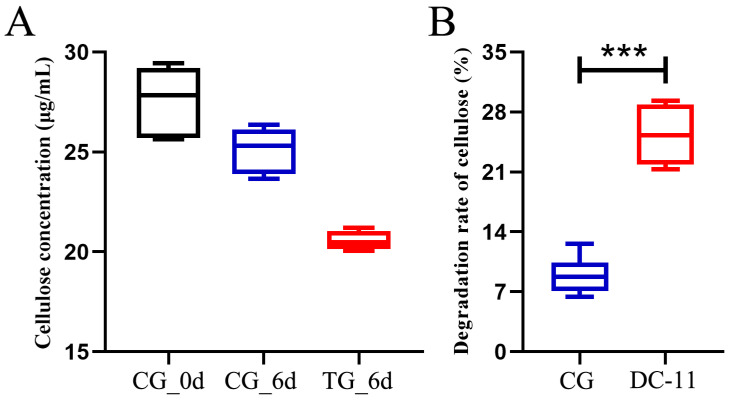
Bioaugmentation of cellulose degradation in silkworm excrement by strain DC-11. Data were calculated as mean ± SD in triplicate measurements. Statistical analysis was performed using Student’s *t*-test (*** *p* ≤ 0.001). (**A**) Change of cellulose concentration, (**B**) Change of cellulose degradation rate. The colors in the figure has no practical meaning.

Unpretreated silkworm excrement was treated with DC-11, and the cellulose degradation rate was monitored for a period of 6 days, as shown in Figure 6A. On day 0, the cellulose concentration in the silkworm excrement was 18.51 μg/mL in CG. After 6 days, the concentrations of cellulose in silkworm excrement in TG and CG were 11.08 and 17.83 μg/mL, respectively. The cellulose degradation rate of TG treated with DC-11 was 39.57%, which was significantly higher than that of CG (10.01%, Figure 6B). These results signify that strain DC-11 can significantly enhance cellulose degradation in silkworm excrement and accelerate the resource utilization of silkworm excrement.

## 4. Discussion

In this study, six cellulose-degrading bacteria were isolated from silkworm excrement. The strain DC-11 with the strongest capability to produce the enzyme was identified as *B. subtilis*, which is consistent with previous studies. Specifically, *Bacillus* has been reported as the preferred genus for the bacterial source of cellulase gene in most studies, although reports on the use of genes from *Actinomycetes* and other genera have also been published [21,29,30]. A previous study reported that out of 20 cellulolytic bacterial isolates, 10 belonged to the *Bacillus* species. Hussain et al. isolated four strains with high cellulose activity from the Egyptian soil environment, all of which belonged to the *Bacillus* genus [1]. Although it is known that *B. subtilis* produces multiple enzymes such as amylase, cellulose, and proteases, there have been few studies on the cellulase enzyme from this species. A previous study demonstrated that various taxa contain cellulolytic bacteria, including *Pseudomonas*, *Actinomycetes*, *Bacillus,* and *Clostridium* [28,31]. Most of these bacteria mainly produce endoglucanase and exoglucanase. Meanwhile, various species from the *Bacillus* genus, including strains of *B. amyloliquefaciens*, *B. subtilis*, and *B. megaterium*, have been found to secrete cellulase [32,33,34]. 

Various cellulose-degrading bacteria have been isolated from different environments. Mokale Kognou et al. isolated six cellulose-degrading bacteria from a mixture of soils, which were identified as *Paenarthrobacter* sp. MKAL1, *Hymenobacter* sp. MKAL2, *Mycobacterium* sp. MKAL3, *Stenotrophomonas* sp. MKAL4, *Chryseobacterium* sp. MKAL5, and *Bacillus* sp. MKAL6 [35]. Harnvoravongchai et al. isolated a thermophilic cellulose- and hemicellulose-degrading bacterium, *Thermoanaerobacterium* sp. R63, from tropical dry deciduous forest soil [6]. Kim et al. isolated a cellulose-degrading bacterium from the sediment; its optimal growth occurred at pH 7.0 and 20 °C [7]. However, few reports have been published on the isolation and cultivation of cellulose-degrading bacteria from silkworm excrement. A previous study has demonstrated that cellulose-degrading bacteria isolated from agricultural waste composting environments could improve the composting efficiency of cellulose-rich agricultural waste [3]. Therefore, it is necessary to screen cellulose-degrading bacteria from cellulose-rich silkworm excrement.

The enzymatic activity of cellulase produced by bacteria is known to depend on the species. For example, Ekperigin et al. reported that the CMCase activities of *Acinetobacter anitratus* and *Branhamella* sp. were 0.48 and 2.57 U/mL, respectively [36]. Moreover, a previous study showed that *Bacillus* sp. can produce cellulases when cultivated at 50 °C, with maximum avicelase (0.83 U/mL) and CMCase (0.29 U/mL) activities being reached when cultured for 120 and 168 h, respectively [28]. In another study, the cellulolytic bacterium, *B. subtilis* SU40, was isolated from an agricultural field. The strain SU40 exhibited maximum cellulase production under optimized culture conditions, i.e., incubation at pH 7.0 and 3 °C for 36 h. The enzyme was purified with a specific activity of 41.15 U/mg on CMC [37]. Furthermore, Gupta et al. conducted a quantitative study on the enzyme production of several cellulose-degrading strains and found that the enzyme-producing strains isolated from the natural environment originally had low enzyme-producing activity, generally ranging from 0.83 U/mL to 6 U/mL, but the enzyme activity could be increased 2–30 times via optimizing the culture conditions and mutating the wild-type strains [14]. The cellulase activity of the DC-11 strain obtained in this study was higher than that of the strains isolated by these researchers.

Previous studies found that fungal cellulase can be produced extracellularly in large quantities, and *Trichoderma* sp. has been shown to exhibit high cellulase enzymatic activity compared with other fungal genera [38]. The crude cellulase activity of *Trichoderma* sp. was reported by Pirzadah to range from 2.85 U/mL to 3.65 U/mL [39]. Moreover, some bacterial strains, such as *Paenibacillus lautus* strain BHU3, have also recently been reported to be highly efficient at cellulase production, with cellulolytic efficiency of 2.1 U/mL [40]. However, the mean CMCase activity of strain DC-11 reached 3.26 U/mL, although its FPase activity was not very high (7.87 ± 0.25 U/mL). The strain certainly has potential for use in cellulase production at an industrial scale after further improvement of the conditions for enzyme production. Considering the CMC degradation phenotype of strain DC-11 and its related cellulase activities, it appears that its degradation relies on the catalytic activities of both CMCase and CXase. The CMCase here was mostly present as endo-1,4-β-D-glucanase, which hydrolyzes CMC-Na into reducing sugars, such as cello-oligosaccharides, cellobiose, and glucose, before the cellobiose was reduced to glucose via β-glucosidase. Therefore, the key enzymes in CMC-Na degradation may be CMCase and CXase in this case. With further optimization, strain DC-11 could become a high-quality strain that produces high levels of cellulase.

The production of fiber-degrading enzymes depends on various growth parameters, including culture time, inoculum concentration, pH, temperature, culture medium additives (carbon and nitrogen sources), aeration, and the presence of various metal ions as activators and inhibitors. In this study, the cellulase production of *B. subtilis* DC-11 peaked after 20 h of culture and decreased after 48 h. This study suggests that enzyme production decreases as the culture time is prolonged beyond the optimum period. Explanations for this observation have previously been given, and a decrease in enzymatic activity with increasing incubation time may be due to the depletion of nutrients and the production of other by-products in the fermentation medium [29,41]. Ariffin et al. also found that the depletion of nutrients in the medium causes bacterial stress, which results in the inactivation of enzyme secretion [11]. According to the growth and other characteristics of the strain, DC-11 remains within the growth stage for a long period of time after 20 h. As bacterial enzymes are most active in the logarithmic phase, we performed a culture for producing cellulase for 20 h in a follow-up experiment to examine the enzyme characteristics. The optimal inoculum concentration of DC-11 was 2%. When the amount of inoculum exceeded the optimum level, the cellulase activity decreased. In the follow-up experiments of this study, the inoculum amount was 2%, at which the dissolved oxygen and nutrients in the medium reached the optimal levels, enabling the DC-11 strain to produce cellulase more effectively. A previous study demonstrated that excessive inoculation leads to excessive bacterial density, resulting in insufficient nutrients and dissolved oxygen, which ultimately limits the growth of bacteria and reduces the capability of producing enzymes [19].

Optimum pH and temperature are considered to be the most important factors for cellulase production. pH is the most important parameter for microbial growth. pH directly influences the enzymatic production and activities of microbes and also impacts the degradation of substrates. The results of this study showed that *B. subtilis* DC-11 had the highest cellulase yield at pH 6.0. In addition, the ability of cellulase to degrade cellulose was stable over a wide pH range (4.5–7.0). In a previous work, it was observed that the cellulose degradation activity of *Bacillus circulans* peaked at pH 8.0, with most bacterial enzymes showing the best activity between pH 5 and 8 [19,22]. An enzyme’s thermal stability is particularly critical for determining whether it can find significant industrial applications. Each microorganism has an optimum temperature for stable enzyme activities. In this study, the isolate DC-11 was able to survive at a temperature of up to 55 °C, which highlights its industrial value. Strain DC-11 is an acidophilic and heat-resistant cellulose-degrading bacterium, the cellulase activity of which peaks at pH 6 and a temperature of 55 °C. This indicates the potential of DC-11 for application in acidic and high-temperature conditions. In fact, many studies have shown that exogenous microorganisms can accelerate the degradation of cellulose during composting as a result of their better environmental tolerance and cellulase-producing ability. The results of this study also show that strain DC-11 can significantly enhance the degradation of cellulose in silkworm excrement during bioaugmentation experiments [26,42]. The rate of cellulose degradation of TG treated with DC-11 was 39.57%, which was significantly higher than that of CG. Therefore, DC-11 has the potential to be included in a microbial inoculum to be applied for the safe and rapid harmless quick composting of silkworm excrement.

## 5. Conclusions

In this study, the acid-resistant thermophilic cellulolytic bacterium *B. subtilis* DC-11 was isolated from silkworm excrement and characterized. It exhibited good CMCase, CXase, and FPase activities. The optimal conditions for cellulolytic activity were inoculum concentration of 2%, temperature of 55 °C, and pH of 6.0. The cellulolytic activity was found to be relatively stable even at 70 °C, and pH 5.0–9.0. At optimum inoculum concentration, pH, and temperature, the activities of CMCase, CXase, and FPase reached 11.91, 20.61, and 15.40 U/mL, respectively. In addition, cellulose degradation using *B. subtilis* DC-11 in silkworm excrement was significantly enhanced during bioaugmentation experiments. The cellulose degradation rate of TG treated with DC-11 was 39.57%, which was significantly higher than that of CG (10.01%). The findings suggest that *B. subtilis* DC-11 has immense economic potential for the efficient resource treatment of cellulose-rich agricultural waste such as silkworm excrement.

## Figures and Tables

**Table 1:** The size of zone of clearance of the isolates.

Isolate’s ID	d (mm)	D (mm)	Cellulolytic Index (D/d)
DC-5	0.40	1.30	3.250
DC-10	0.20	0.50	2.500
DC-11	0.50	1.75	3.500
DC-20	0.50	1.30	2.670
DC-25	0.30	0.70	2.300
DC-28	0.40	1.20	3.000

**Table 2:** Physiological and biochemical identification of the strain DC-11.

Physiological and Biochemical Test	Results
Hydrogen sulfide	+
Gelatin liquefaction	+
Contact enzyme assay	+
Methyl red	−
V-P	+
Indole test	+
Starch hydrolysis	+
Salt tolerance 3% NaCl test	+

Note: “+” is positive, “−” is negative.

## Data Availability

Data sharing not applicable.
